# Supplementary material for: Analysis of cranial tenocyte heterogeneity reveals a role for Wnt signaling in tendon attachments
Source: Development. 2026 Jan 26;153(2):dev205047. doi: 10.1242/dev.205047 (PMC12891946; doi:10.1242/dev.205047)
Supplement: Supplementary information [file develop-153-205047-s1.pdf]

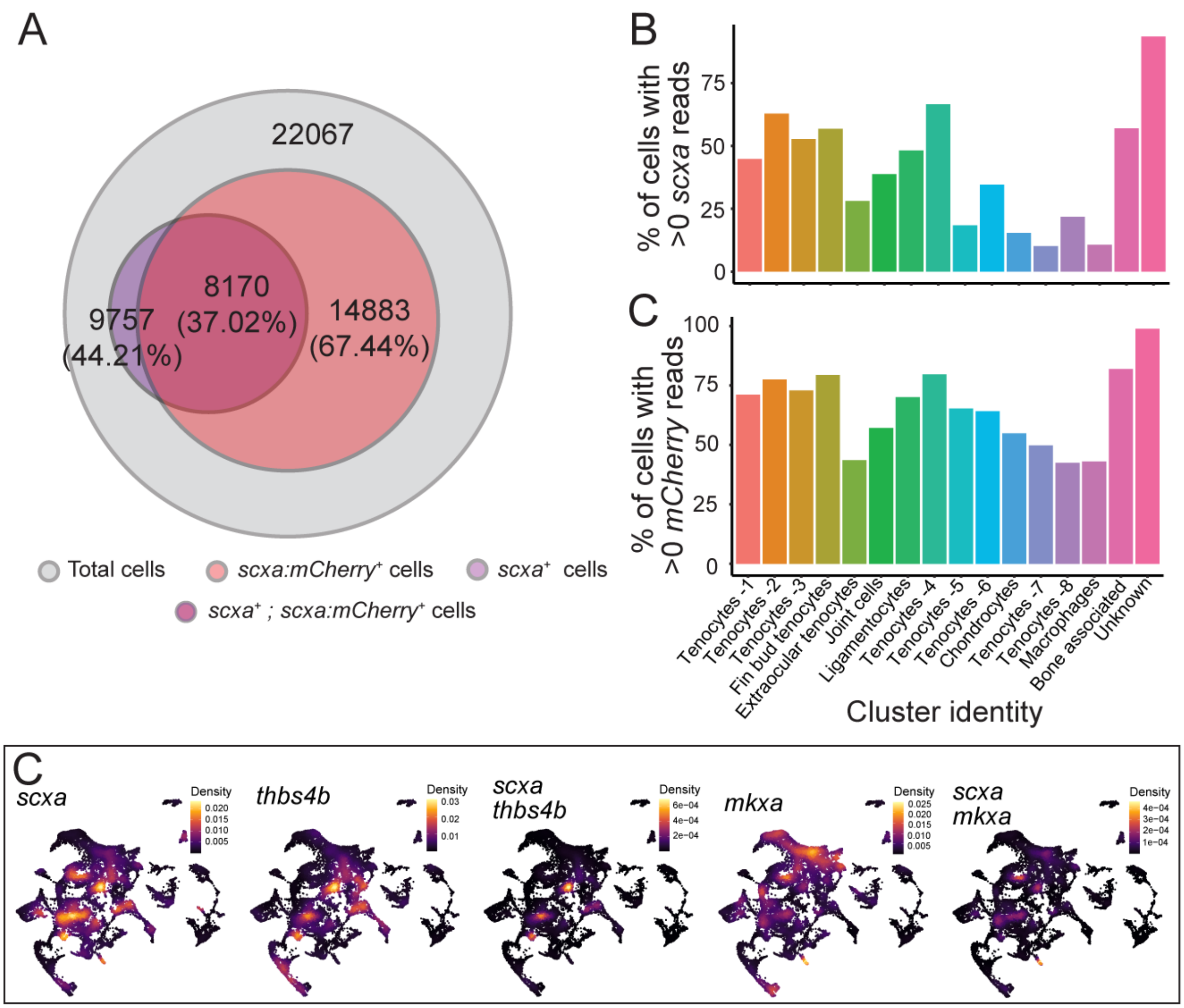

Fig. S1. Quality control of cell sorting and scRNA-seq data reveals tenocyte marker expression distributed across the dataset. (A) Venn diagram displaying proportions of cells within scRNA-seq dataset containing >0 *mCherry* reads, >0 *scxa* reads, and both >0 mCherry and >0 *scxa* reads. (B-C) Bar plot of clusters within scRNA-seq dataset displaying percentage of cells containing > 0 *scxa* reads (B) or >0 *mCherry* reads (C). (D) Nebulosa plots displaying distributions of expression of tenocyte marker genes *scxa, thbs4b, scxa/thbs4b* joint expression, *mkxa*, and *scxa/mkxa* joint expression across UMAP of scRNA-seq dataset.

Development • Supplementary information

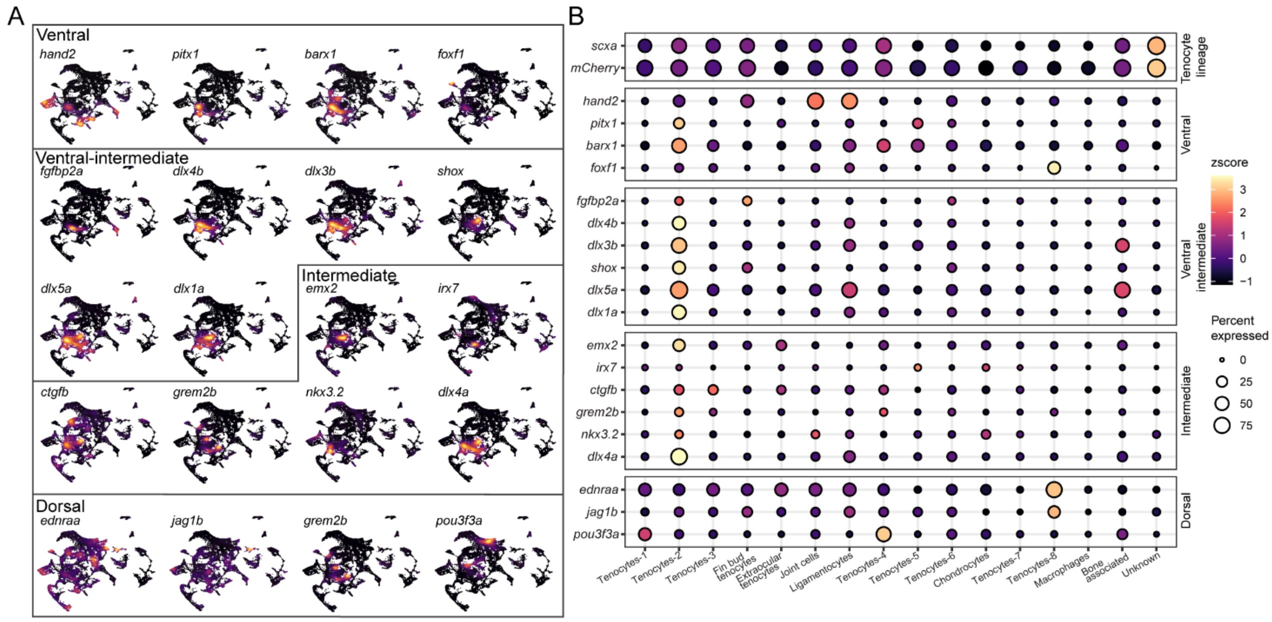

Fig. S2. Patterning genes distinguish tenocytes associated with spatially distinct craniofacial regions. (A) Nebulosa plots displaying expression distributions of ventral, dorsal and ventral-intermediate patterning genes across different clusters that broadly correlate with the locations of craniofacial cartilages of the pharyngeal arches. (B) Dot plot displaying percentages of cells expressing ventral, dorsal, and ventral-intermediate patterning genes across individual clusters.

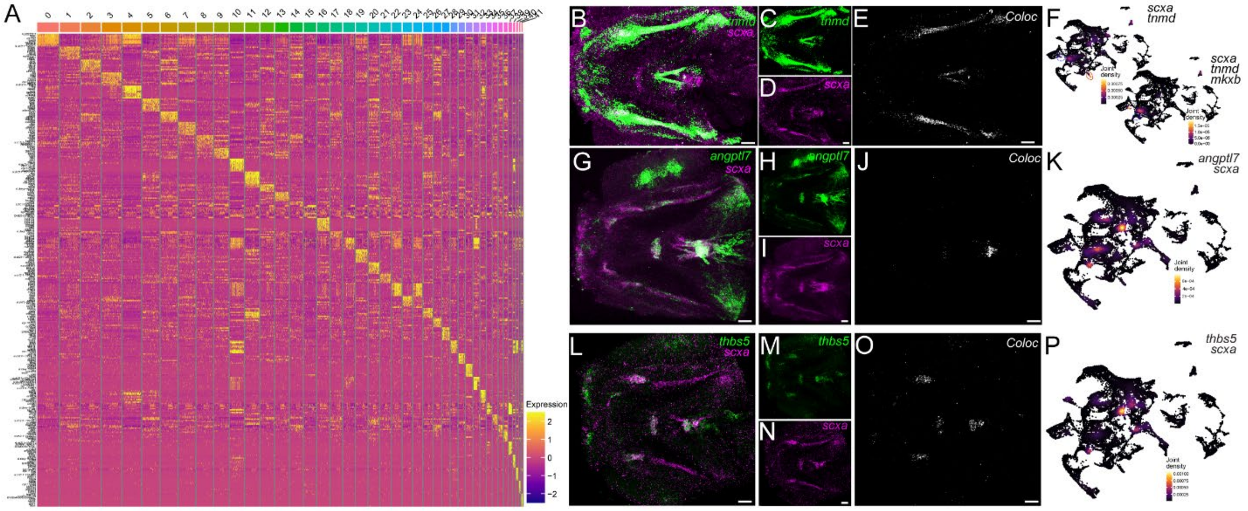

Fig. S3. Cluster-specific expression of differentially expressed genes in cranial tenocytes. (A) Heat map displaying expression of top 10 variable genes in each cluster of UMAP with higher resolution unsupervised clustering shown in Fig. 2 (**Supplementary Table 2**). (B-E, G-J, L-O) Ventral views of cranial tendons at 72 hpf embryos show expression of *scxa*, *tnmd*, *angptl7* and *thbs5* using isHCR across tenocytes and ligamentocytes of various tendons and ligaments. (F, K, P) Nebulosa plots of *tnmd*, *mkxb*, *angptl7*, and *thbs5* co-expressed with *scxa* displaying restricted expression to specific clusters on the UMAP. Scale bars: 30 microns.

Development • Supplementary information

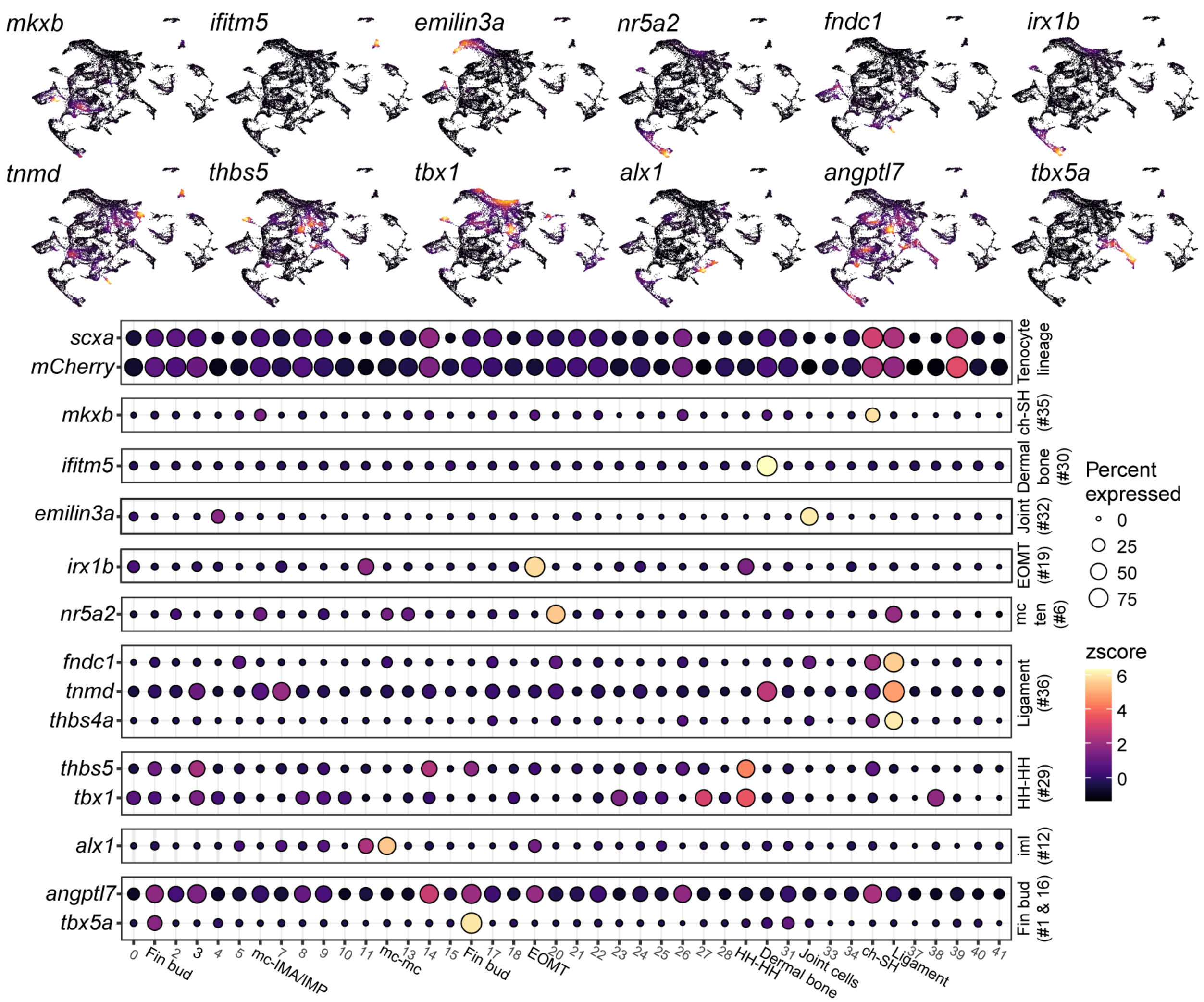

Fig. S4. Cluster-specific expression of isHCR validated marker genes in cranial tenocytes. (A) Nebulosa plots displaying individual expression of isHCR validated marker genes shown in Fig. 2. (B) Dot plot displaying percentage of cells expressing isHCR validated marker genes with high expression across all clusters, marking tenocyte and ligamentocyte populations.

Development • Supplementary information

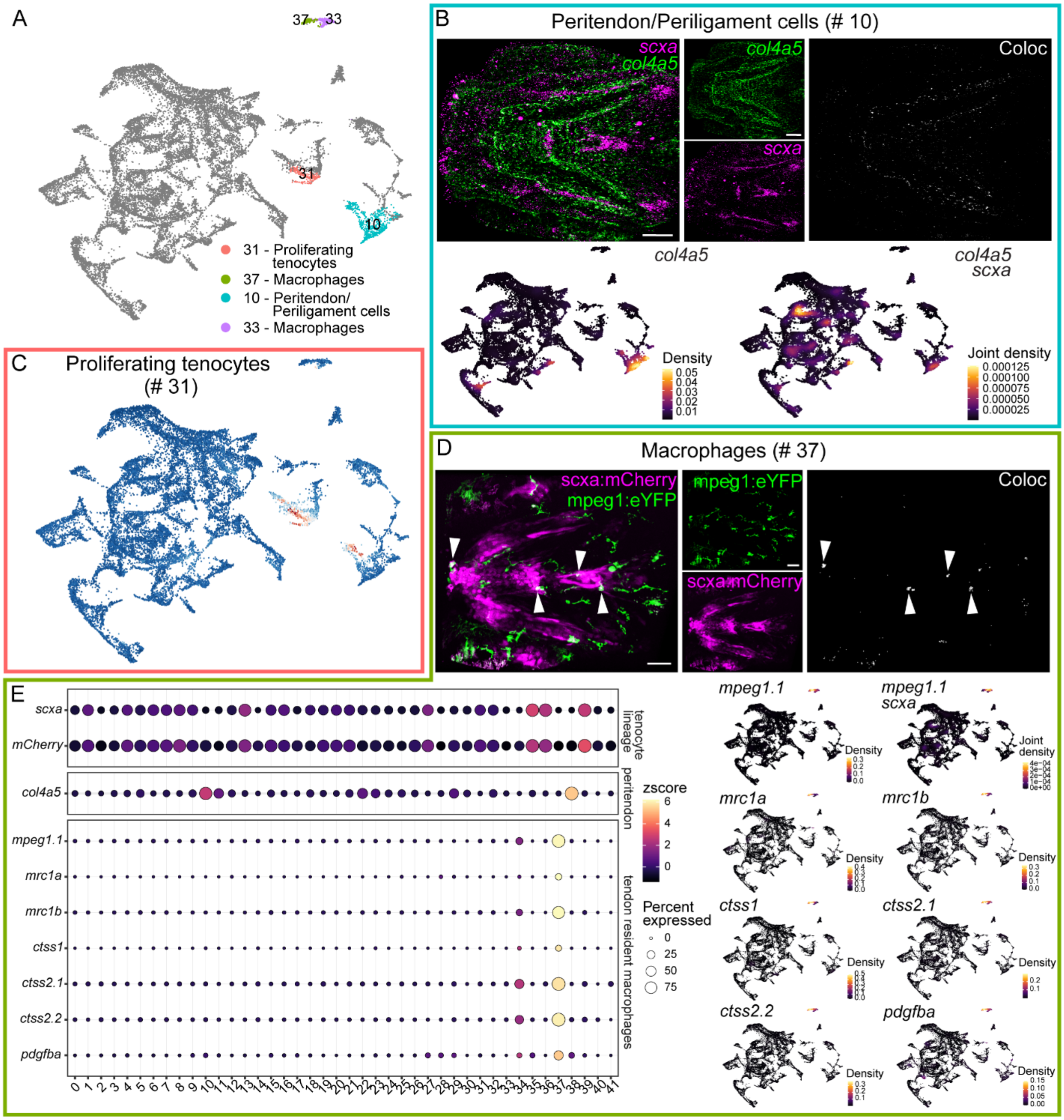

Fig. S5. scRNA-seq analyses reveal peritendon/periligament cells, proliferating tenocytes, and tendon-resident macrophage subpopulations. (A) UMAP plot showing clusters 31, 37, 10 and 33, identified through differentially expressed gene (DEG) analysis as expressing markers characteristic of proliferating tenocytes, macrophages and peritendon or periligament cells. (B) Ventral view of 72 hpf embryo head showing isHCR

Development • Supplementary information

expression of *scxa*, and *col4a5* (a marker from DEG analysis) in cells associated with peritendon and periligament (particularly mc-pq, pq-hs ligaments) regions with colocalization image showing cells coexpressing *col4a5* and *scxa*. Nebulosa plots show heatmaps that reveal clusters of tenocytes coexpressing *col4a5* and *scxa* (cluster 10). (C) Feature plot representing module score of proliferating cell markers (G2/M phase genes, (see Supplementary table 1) with higher score in cells from clusters 31 and subset of cluster 10. (D) Ventral view of a live double transgenic 72 hpf embryo showing tenocytes (scx:mCherry) and macrophages (mpeg1:eYFP) in the developing pharyngeal region. Arrowheads point to macrophages that coexpress *mpeg1* and *scxa* as identified from the Coloc panel. (E) Dot plot and Nebulosa plots show expression of *col4a5* and macrophage related genes (*mpeg1.1, mrc1a, mrc1b, ctss1, ctss2.1, ctss2.2, pdgfba*) across all clusters, displaying cluster-specific expression at clusters 10 and 37 respectively. Scale bars: B – 50 microns, D – 40 microns.

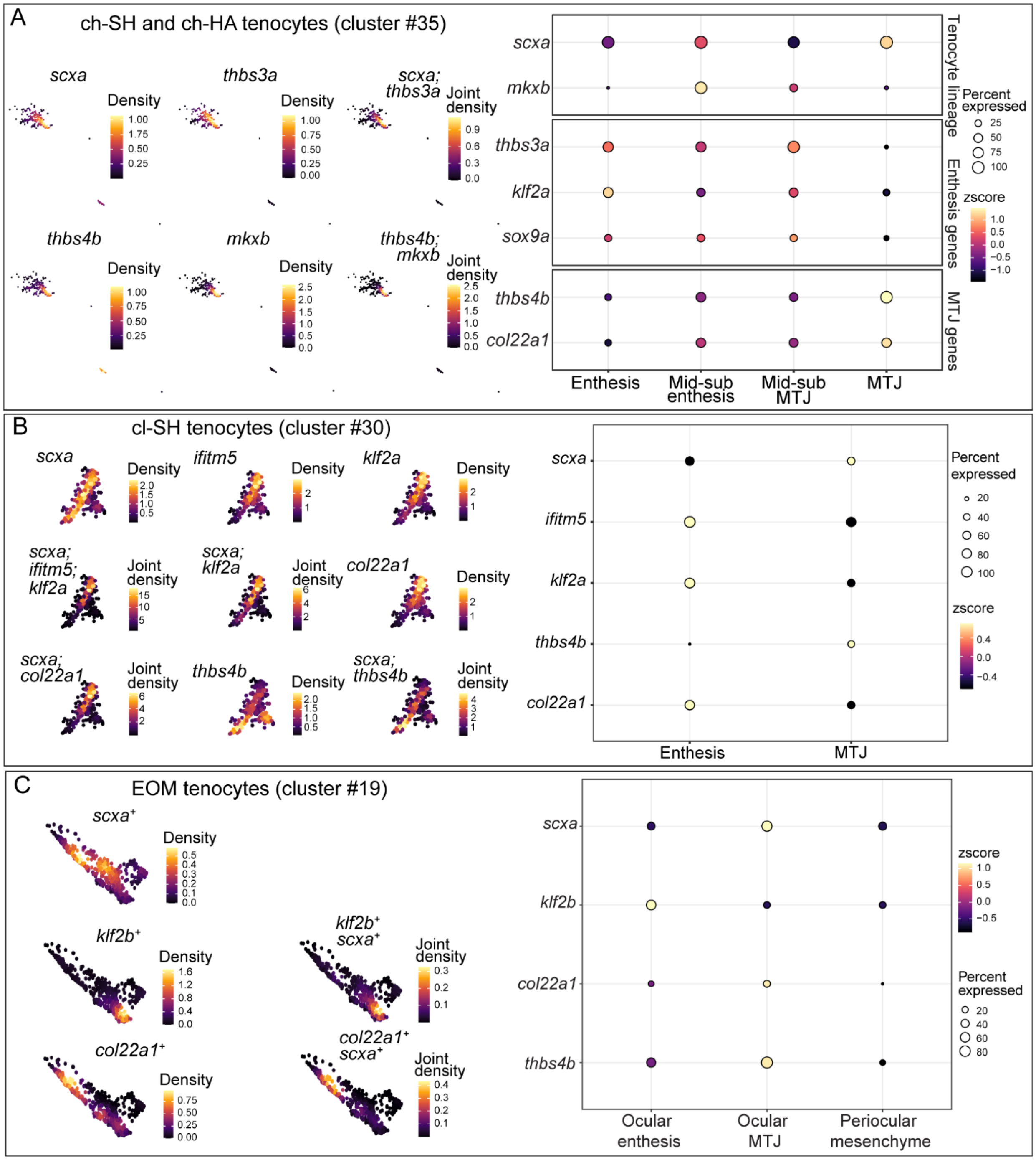

Fig. S6. Subclustering of clusters 35, 30, and 19 reveal distinct MTJ and enthesis expression domains within different tendons.

(A) Nebulosa plots of cluster 35 and dot plots of subclusters of 35 displaying expression distributions of tenocyte markers *scxa*, *mCherry, mkxb* (ch-SH specific) MTJ marker *thbs4b* and

Development • Supplementary information

col22a1, enthesis markers *klf2a, klf2b*, *sox9a, thbs3a* (ch-SH specific). (B) Nebulosa plots of cluster 30 and dot plots of subclusters of 30 displaying expression distributions of tenocyte marker *scxa*, dermal bone marker *ifitm5*, enthesis marker *klf2a*, and MTJ markers *thbs4b* and *col22a1*. (C) Nebulosa plots of cluster 19 and dotplots of subclusters of 19 displaying expression distributions of tenocyte marker *scxa*, enthesis marker *klf2b* (extraocular muscle tenocyte specific), and MTJ markers *thbs4b* and *col22a1*.

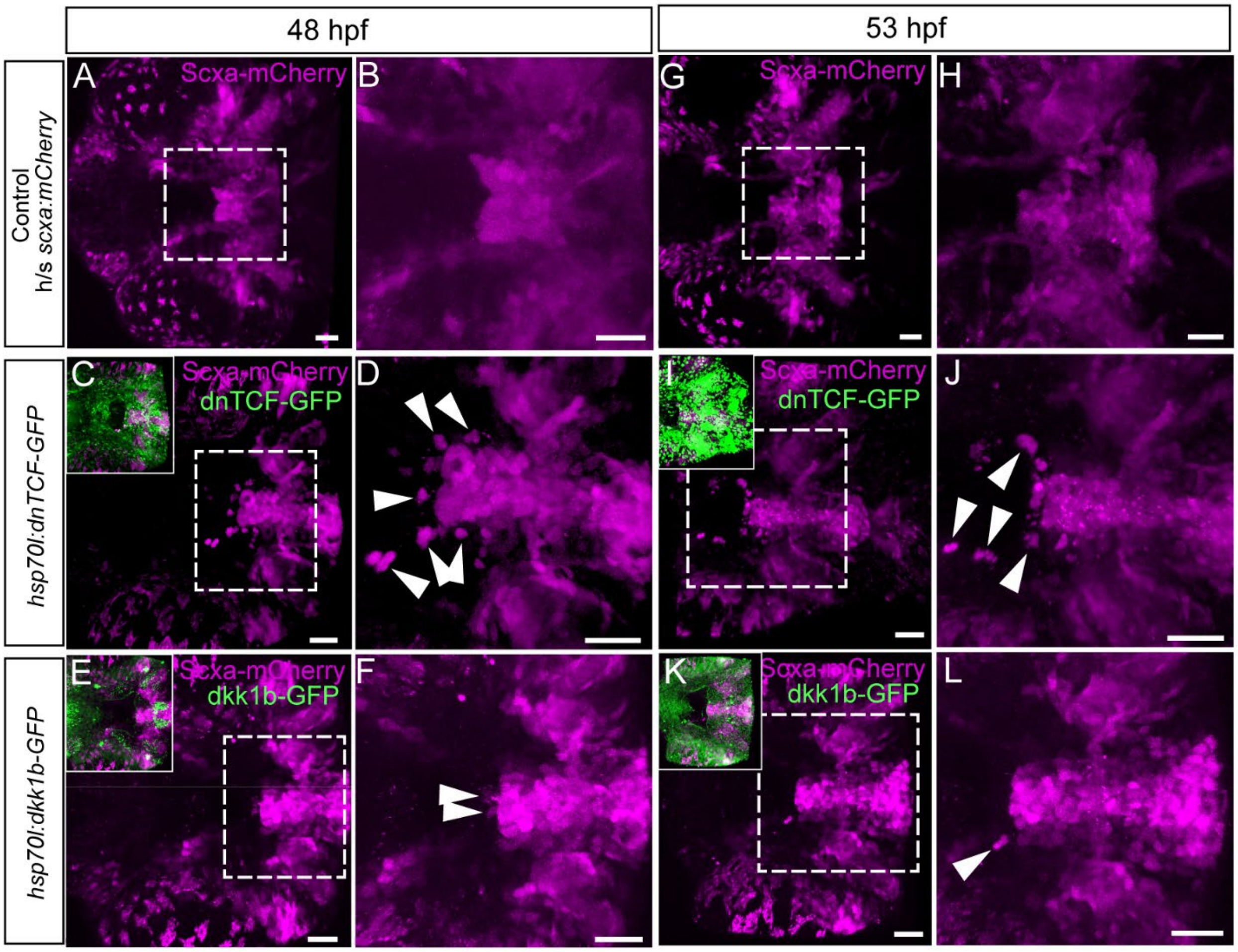

Fig. S7. Genetic perturbation of canonical Wnt signaling disrupts condensation of ventral and medial cranial tenocytes. Live imaging of heat shocked (h/s) control (*Tg(scx:mCherry)*) (A,B,G,H), *hsp70l:dnTF-GFP; scxa:mCherry* (C,D,I,J), *hsp70l:dkk1b-GFP; scxa:mCherry* (E,F,K,L) 48 hpf and 53 hpf embryos showing condensation defects of tenocytes of ventral tenocyte progenitor clusters marked by expression of mCherry. h/s expression was verified by GFP expression shown in insets. Tendon progenitor clusters in ventral-medial regions are outlined by dotted white line in (A, C, E, G, I, K) and magnified in (B, D, F, H, J, L) respectively. Arrowheads point to ectopically migrating/condensing individual tenocyte progenitors. N ~ 5. Scale bars: A,C,G,I,K – 30 microns, E,H – 20 microns, B,D,F,H, - 15 microns, J – 10 microns.

Development • Supplementary information

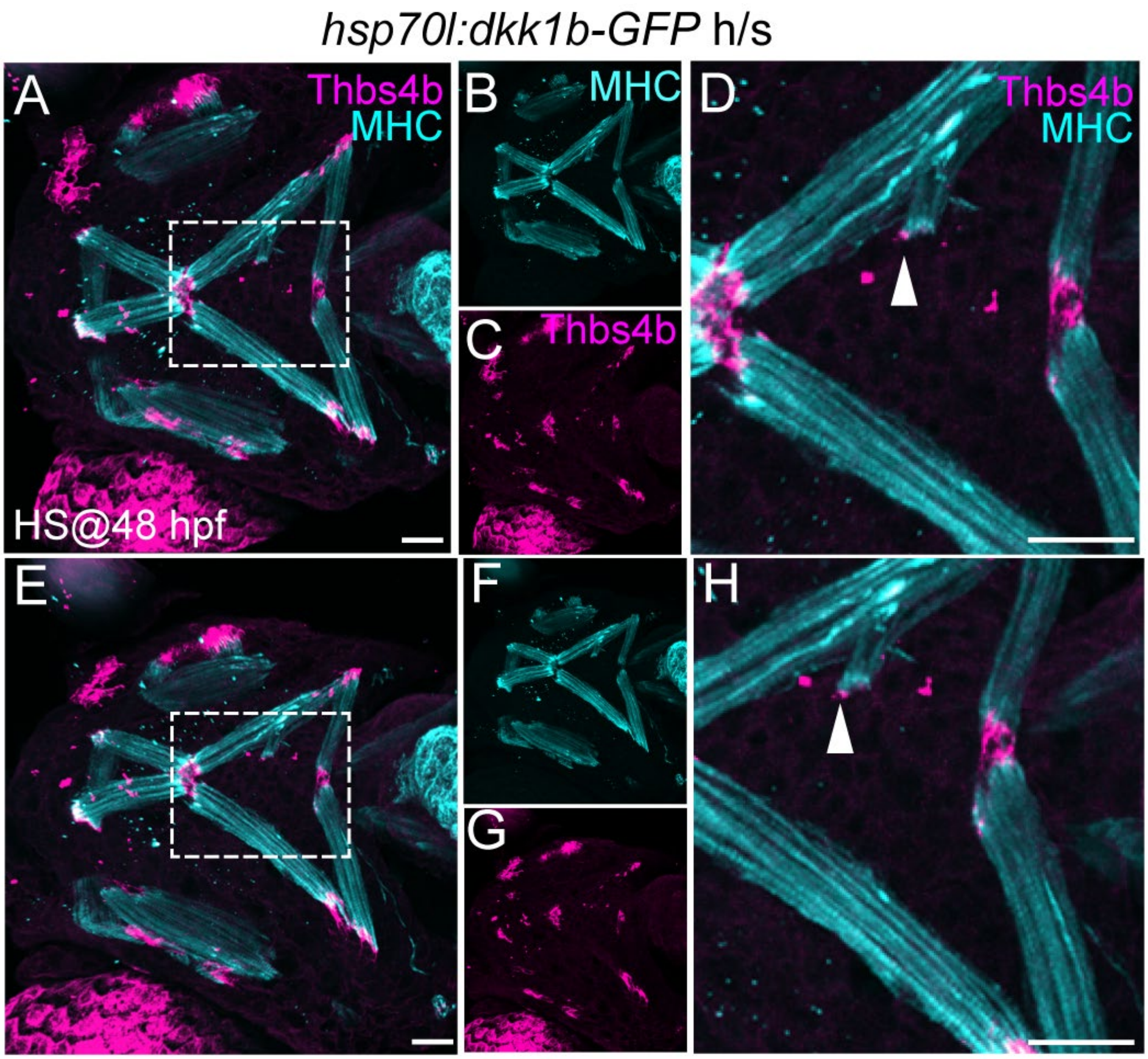

Fig. S8. Genetic downregulation of Wnt signaling causes formation of ectopic myotendinous junctions (MTJs). Ventral (A-D) and ventral-lateral (E-H) views of 72 hpf transgenic (Tg(*hsp70l:dkk1b-gfp*)) embryos immunostained with anti-Thbs4b (MTJ ECM), and anti-MHC (muscles) to visualize tendon and MTJ ECM. Rectangle with dotted white line outlines the region around the interhyal (IH) muscle with ectopic attachments, shown at higher magnification in D,H with arrowhead pointing to Thbs4b localization at an ectopic MTJ. N ~ 10. Scale bars: 30 microns.

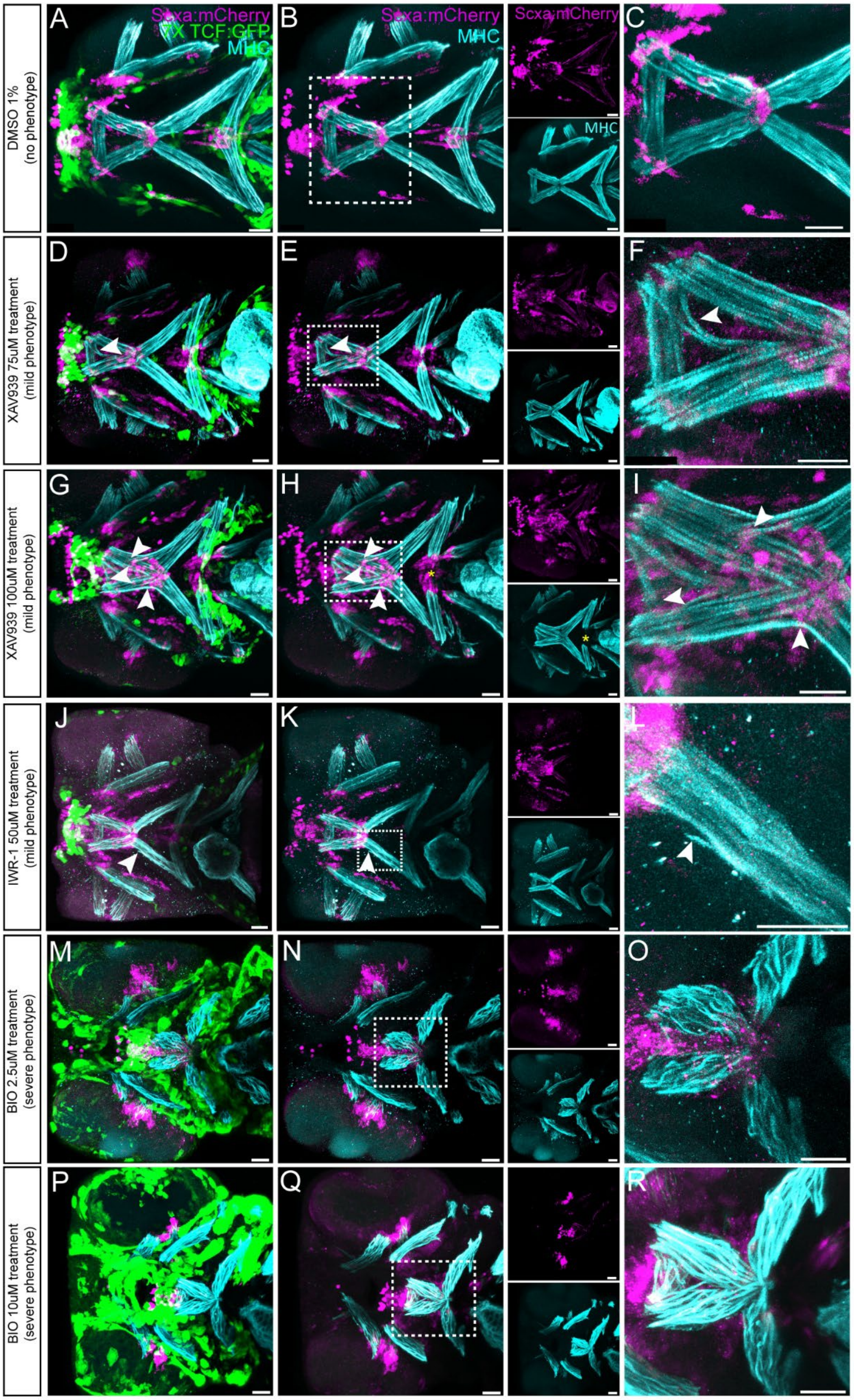

Development • Supplementary information

Fig. S9. Pharmacological perturbations of canonical Wnt signaling cause concentration-dependent cranial muscle attachment defects. Ventral views of 72 hpf double transgenic (Tg(*hsp70l:7XTCF-gfp; scxa:mCherry*)) embryos immunostained with anti-mCherry (tenocytes), anti-MHC (muscles), and anti-GFP (TCF-gfp) to visualize muscle-tendon patterns and responses to Wnt signaling. Control – DMSO carrier (A-C), XAV-939 (75 mM – D-F, 100 mM – G-I), IWR-1 (50 mM – J-L) and BIO (2.5 mM – M-O, 10 mM – P-R) treatments are shown. Rectangle with dotted white lines outlines the region that enlarged in C,F,I, L,O,R and shows representative muscle attachment defects associated with respective treatments. N ~ 80. Scale bars: 30 microns.

Table S1. List of differentially expressed genes from scRNAseq of sorted cranial tenocytes from *Tg(scxa:mCherry)* embryos.

Available for download at
https://journals.biologists.com/dev/article-lookup/doi/10.1242/dev.205047#supplementary-data

Table S2. Differentially expressed genes from high resolution reclustering of scRNAseq dataset of cranial tenocytes.

Available for download at
https://journals.biologists.com/dev/article-lookup/doi/10.1242/dev.205047#supplementary-data

Table S3. Probes/amplifier combinations used in this study

1	*scxa* (NCBI ref # NM_001083069) in B2 with B2 Alexa Fluor 546
2	*klf2a* (NCBI ref # NM_131856.3) in B3 with B3 Alexa Fluor 647
3	*thbs4b* (NCBI ref # NM_173226) in B3 with B3 Alexa Fluor 647
4	*nr5a2* (NCBI ref # NM_ 001313729.1) in B1 with B1 Alexa Fluor 488
5	*tbx1* (NCBI ref # NM_183339.1) in B1 with B1 Alexa Fluor 488
6	*thbs5* (NCBI ref # NM_001326350.2) in B3 with B3 Alexa Fluor 647
7	*col4a5* (NCBI ref # NM_001123230.1) in B3 with B3 Alexa Fluor 647
8	*irx1b* (NCBI ref # NM_131823.1) in B1 with B1 Alexa Fluor 488
9	*tnmd* (NCBI ref # NM_001114413.1) in B1 with B1 Alexa Fluor 488
10	*fndc1* (NCBI ref # XM_003200712.5) in B3 with B3 Alexa Fluor 647
11	*emilin3a* (NCBI ref # XM_021474148.1) in B1 with B1 Alexa Fluor 488
12	*col22a1* (NCBI ref # NM_001291376) in B3 with B3 Alexa Fluor 647
13	*thbs3a* (NCBI ref # NM_173225) in B1 with B1 Alexa Fluor 488
14	*angptl7* (NCBI ref # NM_001006073) in B1 with B1 Alexa Fluor 488
15	*klf2b* (NCBI ref # NM_131857) in B1 with B1 Alexa Fluor 488
16	*mkxb* (NCBI ref # NM_001114409) in B3 with B3 Alexa Fluor 647
17	*col4a5* (NCBI ref # NM_001123230.1) in B3 with B3 Alexa Fluor 647
18	*alx1* (NCBI ref # NM_001045074) in B3 with B3 Alexa Fluor 647
19	*ifitm5* (NCBI ref # NM_001177312) in B1 with B1 Alexa Fluor 488

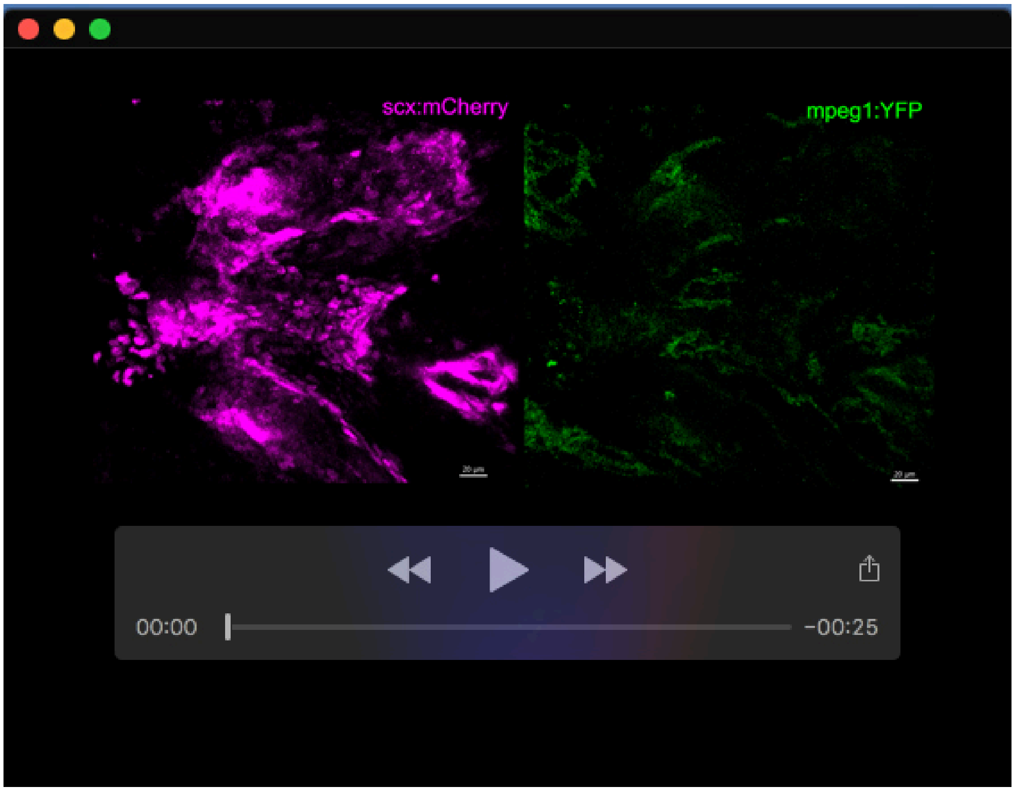

Movie 1. 24 hour Timelapse of 48hpf *Tg(scxa:mCherry;mpeg1:eYFP)* embryo

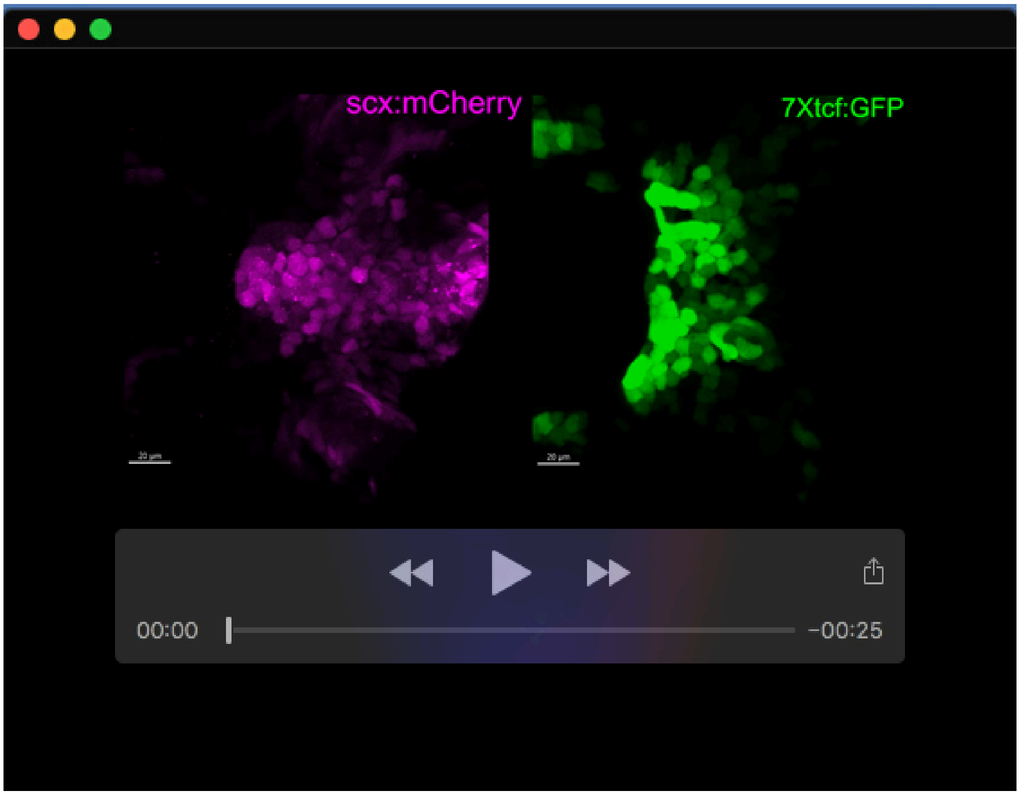

Movie 2. 12 hour timelapse of 48hpf *Tg(scxa:mCherry;7Xtcf:gfp)* embryo.

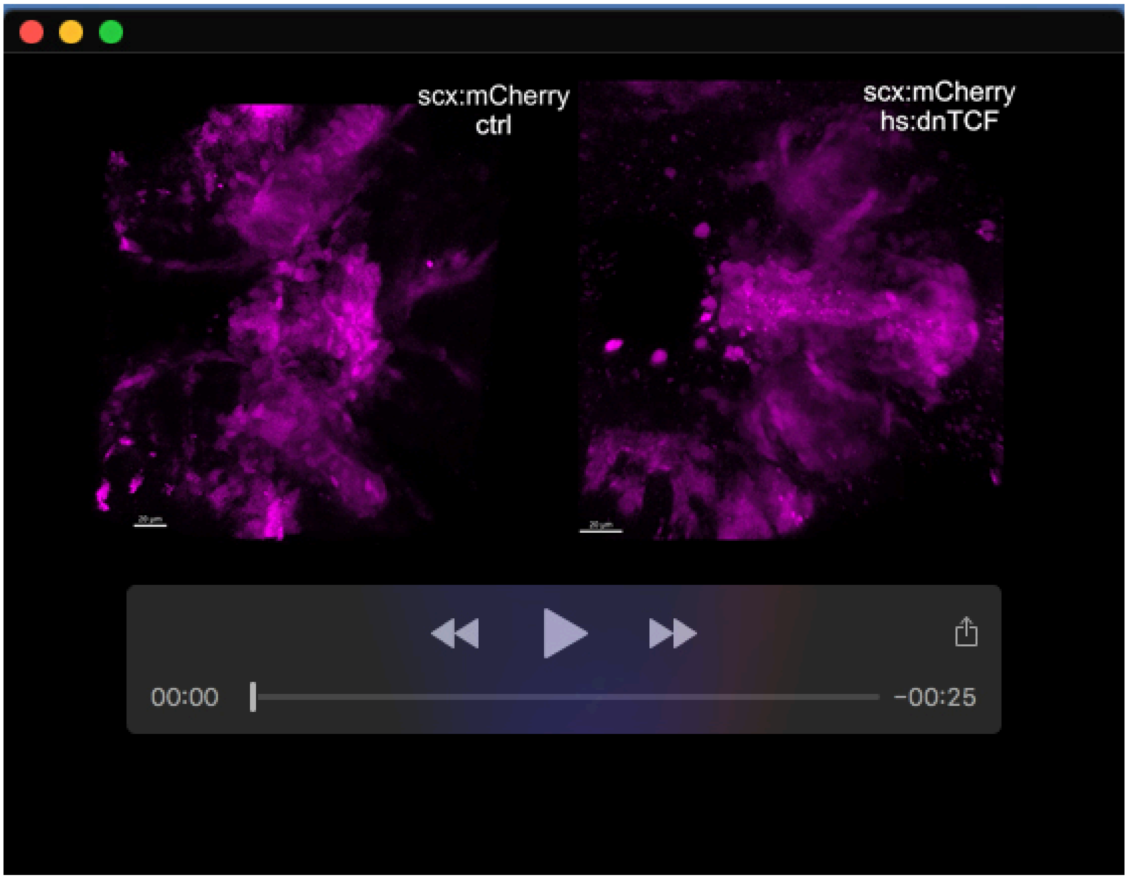

Movie 3. 12 hour timelapse of 48hpf heatshocked *Tg(scxa:mCherry;hsp70l:dnTCF-GFP)* displaying tenocyte aggregation defects

Development • Supplementary information
